# Sustaining stakeholder engagement for health research during the COVID-19 pandemic: Lessons from the RESPIRE programme in Bangladesh, India, Malaysia, and Pakistan

**DOI:** 10.7189/jogh.12.03057

**Published:** 2022-09-03

**Authors:** Genevie Fernandes, Tracy Jackson, Aaliyan Kashif, Ahmed Ehsanur Rahman, Ajay Kumar Roy, Ashraful Islam ASMD, Biswajit Paul, Dhiraj Agarwal, Fahmeda Akter, Farishtey Muanka, GM Monsur Habib, Hana Mahmood, Harsh Regi, Himangi Lubree, Jayakayatri Jeevajothi Nathan, Osman Mohammad Yusuf, Ramsha Tariq Baig, Rita Isaac, Rutuja Patil, Sabrina Jabeen, Salahuddin Ahmed, Mohammad Shahidul Islam, Sanjay Juvekar, Siân Williams, RESPIRE Collaboration

**Affiliations:** 1 NIHR Global Health Research Unit on Respiratory Health (RESPIRE), Usher Institute, University of Edinburgh, Edinburgh, UK; 2 Neoventive Solutions, Islamabad, Pakistan; 3 icddr,b, Dhaka, Bangladesh; 4 Bangladesh Primary Care Respiratory Society, Khulna, Bangladesh; 5 Projahnmo Research Foundation, Dhaka, Bangladesh; 6 Rural Unit for Health and Social Affairs, Christian Medical College, Vellore, India; 7 Vadu Rural Health Program, KEM Hospital Research Centre, Pune, India; 8 Department of Primary Care, Faculty of Medicine, University of Malaya, Kuala Lumpur, Malaysia; 9 The Allergy and Asthma Institute, Islamabad, Pakistan; 10 Child Health Research Foundation, Dhaka, Bangladesh; 11 International Primary Care Respiratory Group

Governments worldwide responded to the continual waves of the COVID-19 pandemic with unprecedented measures ranging from social distancing and cancellation of public events to border restrictions and complete closure of all but essential services [1]. Health research studies were suspended, becoming casualties of COVID-19-related lockdowns and distancing policies [2]. Engaging and involving stakeholders including patients, community actors, health care providers, and policymakers in research also became challenging as in-person activities were paused or cancelled owing to the risk of SARS-CoV-2 transmission [3]. Healthcare providers were pulled into frontline care. Policymakers shifted priority from pressing public health issues including other respiratory health conditions towards COVID-19 [4].

While several reports have discussed the impact of the COVID-19 pandemic on health research studies and how teams have responded [2], few have captured the effects of pandemic control measures on stakeholder engagement within research [3]. We report our experiences from the NIHR Global Health Research Unit on Respiratory Health (RESPIRE) funded by the UK National Institute of Health Research (NIHR) in 2016-2017, a partnership between the University of Edinburgh in the United Kingdom, International Primary Care Respiratory Group, and ten research partners across Bangladesh, India, Malaysia, and Pakistan [5]. We describe how RESPIRE researchers responded to the pandemic and adapted their approach to sustain engagement with a wide range of stakeholders.

## ADAPTED APPROACH FOR SUSTAINING STAKEHOLDER ENGAGEMENT

### Identifying and addressing stakeholders’ needs and concerns

Through informal consultations via telephone calls, virtual platforms and, where government guidelines permitted, in-person meetings, partners identified and addressed the needs and concerns of each stakeholder group in light of the pandemic, as shown in **Table 1**. Patients, caregivers, and community members expressed fear of getting infected in health facilities and highlighted new barriers, like travel difficulties due to the suspension of public transport, increased burden of unemployment, as well as existing challenges with accessing the internet and digital platforms. Community health workers, health care providers and public health managers reported working long hours on COVID-19 with limited time for any engagement and concern about the potential risk of infection by bringing together patient and community groups. Policymakers and clinical leaders focused their resources on pandemic response, reducing their availability to engage with RESPIRE researchers.

**Table 1:** Addressing the needs and concerns of key stakeholder groups during the COVID-19 pandemic

Stakeholder group	Needs and concerns	Response
Patients, caregivers, family members, local community leaders and members	• Fear of getting infected with COVID-19 at the health facility (venue for stakeholder meetings) • Suspension of public transport during lockdowns making travel challenging • Hit in income • Loss of daily wages for informal workers on attending any meetings or events • Lack of access to desktop/laptop, smartphones, and internet connection • Unaffordability of internet costs • Difficulty in using digital technology	• Maintained rapport through consistent communication • Identified lead liaison for patient and public involvement groups • Distributed informational pamphlets in local communities to raise awareness about COVID-19, including preventive and care-seeking behaviours • Organized meetings in local community venues to avoid any travel • Ensured infection prevention and control measures during in-person meetings • Invited local leaders to small group in-person meetings, who then disseminated information to patients and community members • Provided mobile internet costs for virtual engagement activities
Community health workers, health care providers, and public health managers	• Risk of COVID-19 infection • Lack of time due to increased workload from COVID-19 responsibilities	• Ensured infection prevention and control by providing masks, gloves, and sanitisers, and conducting workshops to discuss and encourage the practice of safety measures • Organized virtual engagement meetings to reduce time requirement • Conducted capacity-building workshops on virtual platforms
Policymakers (provincial and central government officials) and clinical leaders (professional medical associations)	• Priority and attention shifted towards COVID-19 response • Challenges in scheduling meetings due to COVID-19 • Perception that virtual meetings as against face-to-face discussions were impersonal and sometimes discourteous	• Scheduled regular communication and in-person visits, where possible, to retain visibility and maintain rapport and trust • Used policy hooks for influence and to increase the relevance of research study and findings to the current policy issues • Identified and worked closely with champions or interested and influential individuals in the policy-making environment, who supported the cause, ensured the buy-in of allied stakeholders, and helped in addressing any barriers

### Practising and promoting risk reduction

COVID-19 risk reduction measures have been at the heart of engagement with all stakeholder groups. Neoventive Solutions, a RESPIRE partner in Pakistan, trained community-based lady health workers on childhood pneumonia management in a ventilated indoor space using face masks and sanitisers, maintaining six feet distance between participants, and monitoring body temperature. Similarly, the Vadu Rural Health Program, KEM Hospital Research Centre, a partner in India, conducted 156 meetings in 120 villages with community leaders and health workers in small groups in open-aired spaces such as the temple and ventilated indoor venues like the public library [6]. As part of their study testing the feasibility of introducing pulse oximetry in routine child health services, icddr,b (previously known as International Centre for Diarrhoeal Disease Research, Bangladesh) trained public health care providers and managers in infection prevention and control measures and provided them masks, gloves and sanitisers.

### Increased use of media, social media, and virtual platforms

Partners increasingly appeared on prime-time health programmes on television and radio, contributed to informational articles in newspapers, distributed information leaflets in local communities, and published in peer-reviewed journals. Child Health Research Foundation and Bangladesh Primary Care Respiratory Society, partners from Bangladesh, appeared regularly in mainstream electronic, print, and social media channels, using COVID-19 as an entry point to discuss respiratory health and RESPIRE findings. Child Health Research Foundation also employed the mid-media route through “pneumonia corners” (informational kiosks) set up outside public health facilities to engage with parents and caregivers of children <5 years on preventive and health care-seeking behaviours.

**Figure:**
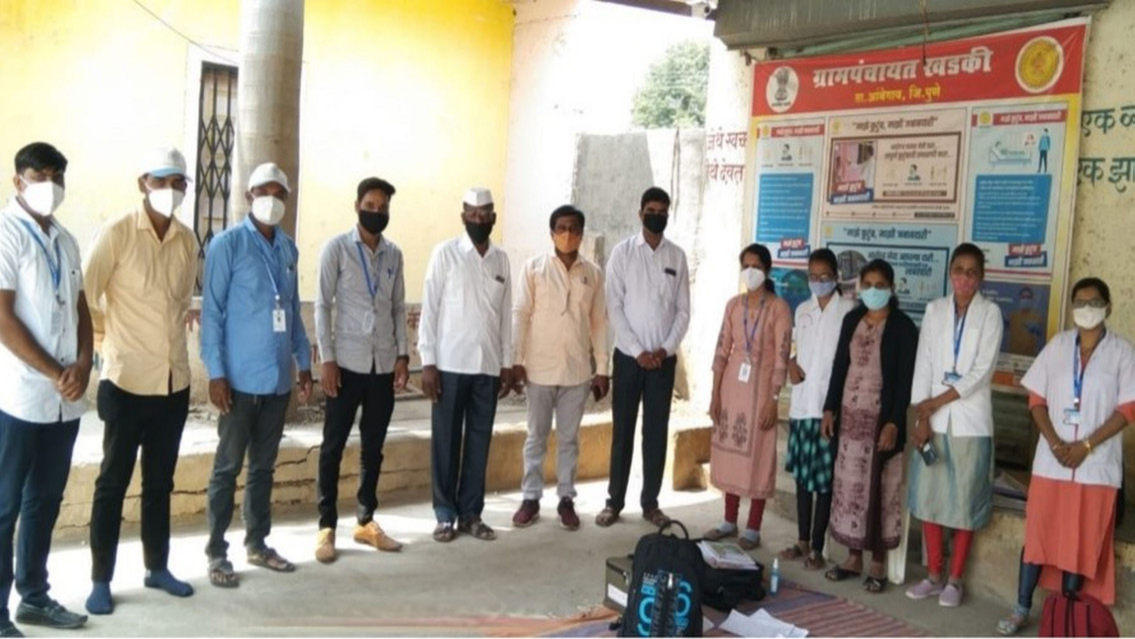
Photo: KEMHRC team conduct a meeting with the village leaders and community representatives to brief them about the RESPIRE serosurveillance study, which resulted in community support for successful recruitment and data collection amidst COVID-19-related restrictions. Source: Copyright-free image provided by one of the co-authors (HL) from Vadu Rural Health Program, KEM Hospital Research Centre, Pune, India.

Action messages emerging from RESPIRE research were disseminated in text, audio, and video formats on YouTube, Facebook, Instagram, and WhatsApp, generating public visibility, awareness, and engagement. Based on consultations with patients and caregivers, the Allergy and Asthma Institute, a partner in Pakistan, developed a documentary and an animation film to raise awareness about pollen allergy, challenges faced by patients and their families, and encourage appropriate care seeking behaviours [7]. Several partners also employed virtual platforms such as Zoom and Microsoft Teams to conduct capacity building with primary health care providers, seminars with clinical leaders, and dissemination workshops with policy makers. The University of Malaya and Universiti Putra Malaysia invited individuals living with asthma and primary health care providers to a design workshop on Microsoft Teams for developing and testing a feasible prototype mobile application for asthma self-management.

### Phased engagement

Partners phased their engagement based on the evolving government guidelines for COVID-19 control and stakeholder capacities. The Rural Unit for Health and Social Affairs in India initially resumed community engagement with small group meetings. As COVID-19 cases reduced and restrictions eased, the team moved to audio-visual screenings for relatively larger groups in big cross-ventilated wedding halls and community spaces and arranged for community leaders to visit their facility for health promotion training. Responding to feedback from patients living with chronic obstructive pulmonary disease, the Bangladesh Primary Care Respiratory Society initially offered advice over telephone calls for continuing their home-based pulmonary rehabilitation by proceeding to small group meetings in their clinic with masking and physical distancing among other prevention measures.

### Consistent communication

Staying in touch with stakeholders and consistently communicating, even without planned agendas, is key for retaining visibility and trust. Projahnmo Research Foundation, a partner in Bangladesh, credits regular communication through telephone calls, in-person visits where possible, and virtual meetings for the increased interest and commitment from stakeholders from the Ministry of Health and Family Welfare. Through consistent communication, the Projahnmo team also identified interested and influential individuals from the Ministry of Health and Family Welfare and used their support to marshal buy-in from other policy stakeholders for their study testing digital auscultation for diagnosing childhood pneumonia in communities.

## IMPACT OF THE ADAPTED APPROACH

Partners reported greater receptiveness and raised levels of awareness among community members about COVID-19 and respiratory health-related knowledge and behaviours being promoted under RESPIRE. The Vadu Rural Health Program credited community engagement for the recruitment of 14 500 participants and successful data collection for their COVID-19 serosurveillance study, increased uptake of a pulmonary rehabilitation service, and sustained public trust. Partners also observed increased participation of community health workers and health care providers for training and capacity building, mainly through virtual platforms like Zoom, but also in small group in-person meetings where appropriate. Increased government collaboration was a major outcome evident in participation invitations for national-level consultations for newborn health policy planning in Pakistan, approvals for resuming data collection in India, support for scaling up a larger study, and integrating RESPIRE research findings into national child health guidelines and service delivery in Bangladesh [8].

## CHALLENGES FACED

Many RESPIRE researchers were respiratory clinicians diverted to frontline care or had to venture into local communities for data collection, which increased their risk of exposure to SARS-CoV-2. Given the suspension of planned activities and move towards virtual engagement, it was initially challenging to utilise allocated budgets. While virtual platforms offered a large reach and saved time and costs, they offered limited scope for interactivity compared to in-person engagement. Participants would turn their cameras and sound off during virtual meetings; and activities such as the demonstration of inhalers and spirometers, often best suited to live demonstrations, were challenging to conduct online. Many community members, especially from rural areas, lack smartphones and internet connectivity, cannot afford mobile internet costs, find virtual platforms difficult to use; making such populations less likely to be heard. Diversion towards pandemic response made government policy makers less available; departmental transfers of civil servants caused setbacks, with partners having to restart conversations.

## LESSONS LEARNT

Even during a global public health emergency like COVID-19, stakeholder engagement in health research can be endured with adaptations. We considered risk reduction in every step, consulted, understood, and responded to the needs and concerns of stakeholders, and employed relevant and effective communication channels. Identifying and monitoring stakeholders who are resource-poor and vulnerable is critical so that additional resources and support can be planned and allocated to ensure that no one gets left behind. Contingency plans need to be developed, including flexible allocation of resources, for potential public health emergencies.

Increasing the relevance of research findings to current policy issues and finding synergies with other health priorities is key to garnering policymakers’ interest, trust, and engagement. This lesson remains relevant even in a post-pandemic world, along with other adaptations such as increased public engagement via media channels, capacity building for health care providers through virtual platforms, and consistent communication with stakeholders. Although newer technologies for contact and dialogue were helpful, their effectiveness needs to be explored. Finally, stakeholder engagement cannot be a one-off activity; it must be institutionalised to build longstanding partnerships and close the gap between research production and use. Institutionalisation requires high-level commitment, leadership, and dedicated human and financial resources to plan, implement, monitor, and evaluate stakeholder engagement across all research projects.
